# Invasive Extravillous Trophoblasts Restrict Intracellular Growth and Spread of *Listeria monocytogenes*

**DOI:** 10.1371/journal.ppat.1002005

**Published:** 2011-03-03

**Authors:** Varvara B. Zeldovich, Jennifer R. Robbins, Mirhan Kapidzic, Peter Lauer, Anna I. Bakardjiev

**Affiliations:** 1 Department of Pediatrics, University of California, San Francisco, San Francisco, California, United States of America; 2 Program in Microbial Pathogenesis and Host Defense, University of California, San Francisco, San Francisco, California, United States of America; 3 Biomedical Sciences Program, University of California, San Francisco, San Francisco, California, United States America; 4 Department of Biology, Xavier University, Cincinnati, Ohio, United States of America; 5 Institute for Regeneration Medicine, Human Embryonic Stem Cell Program, Department of Obstetrics and Gynecology, University of California, San Francisco, California, United States of America; 6 Aduro Biotech, Berkeley, California, United States of America; The Rockefeller University, United States of America

## Abstract

*Listeria monocytogenes* is a facultative intracellular bacterial pathogen that can infect the placenta, a chimeric organ made of maternal and fetal cells. Extravillous trophoblasts (EVT) are specialized fetal cells that invade the uterine implantation site, where they come into direct contact with maternal cells. We have shown previously that EVT are the preferred site of initial placental infection. In this report, we infected primary human EVT with *L. monocytogenes*. EVT eliminated ∼80% of intracellular bacteria over 24-hours. Bacteria were unable to escape into the cytoplasm and remained confined to vacuolar compartments that became acidified and co-localized with LAMP1, consistent with bacterial degradation in lysosomes. In human placental organ cultures bacterial vacuolar escape rates differed between specific trophoblast subpopulations. The most invasive EVT—those that would be in direct contact with maternal cells *in vivo*—had lower escape rates than trophoblasts that were surrounded by fetal cells and tissues. Our results suggest that EVT present a bottleneck in the spread of *L. monocytogenes* from mother to fetus by inhibiting vacuolar escape, and thus intracellular bacterial growth. However, if *L. monocytogenes* is able to spread beyond EVT it can find a more hospitable environment. Our results elucidate a novel aspect of the maternal-fetal barrier.

## Author Summary

Infection of the placenta and fetus is an important cause of pregnancy complications and fetal and neonatal morbidity and mortality. *Listeria monocytogenes* is an intracellular bacterial pathogen that causes pregnancy-related infections in humans. The pathogenesis of listeriosis during pregnancy is poorly understood. We have previously shown that transmission of *L. monocytogenes* from maternal cells and tissues to fetal cells occurs in the uterine implantation site, and that a small subpopulation of specialized fetal cells called extravillous trophoblasts are the preferred initial site of infection. Here we use primary human placental organ and cell culture systems to characterize the intracellular fate of *L. monocytogenes* in extravillous trophoblasts. We found that these cells entrap bacteria in vacuolar compartments where they are degraded and therefore reduce bacterial dissemination into deeper structures of the placenta. Our study provides new insights into the nature of the maternal-fetal barrier. Extravillous trophoblasts that are accessible to infection with intracellular pathogens from infected maternal cells have host defense mechanisms that constitute a bottleneck in maternal-fetal transmission.

## Introduction

*L. monocytogenes* is a ubiquitous, facultative intracellular, Gram-positive bacterium that causes food-borne disease in humans and other mammals [1], [2]. Humans are exposed relatively frequently to *L. monocytogenes*: healthy adults in the United States are estimated to ingest 10^5^ bacteria at least four times per year [3]. Ingestion of *L. monocytogenes* by an immunocompetent host is relatively innocuous, but in immunocompromised individuals and pregnant women listeriosis is a severe disease [1], [4]. In the US there are ∼530 cases per year of listeriosis during pregnancy (FDA, 2009). The clinical manifestations depend on the gestational age. During the second trimester *L. monocytogenes* is the cause of ∼3% of spontaneous abortions [5], [6]. Infection around term results in neonatal disease with mortality of up to 50% [7]. The mechanisms by which *L. monocytogenes* infects the placenta and crosses the maternal-fetal barrier are controversial and still poorly understood.

The intracellular life cycle of *L. monocytogenes* has been characterized in a variety of different cell lines as well as primary murine bone marrow-derived macrophages [8], [9]. *L. monocytogenes* is taken up either by phagocytosis or internalized via interaction of bacterial surface proteins, such as internalin A (InlA), with host cell receptors, such as E-cadherin [10], [11]. After internalization, the bacterium finds itself in an endocytic vacuole that develops into a late endosome and acidifies slightly [12]. Acidification activates the pore-forming toxin listeriolysin O (LLO) that is important for escape of the bacterium into the host cytosol, where *L. monocytogenes* replicates rapidly [13], [14]. The listerial protein ActA nucleates actin and allows *L. monocytogenes* to spread from cell-to-cell without exposure to the extracellular milieu [15].

The placenta has to protect the fetus from vertical transmission of pathogens while also providing an environment of immunological tolerance for the fetal allograft [16]. How the placenta accomplishes these contradictory tasks is unknown. It has long been postulated that the placenta is an immune-privileged organ that has diminished adaptive immune defenses in order to establish tolerance. However, low placental infection rates in the face of frequent pathogen exposure suggest that the organ itself must have defense mechanisms against infection.

**Figure 1:**
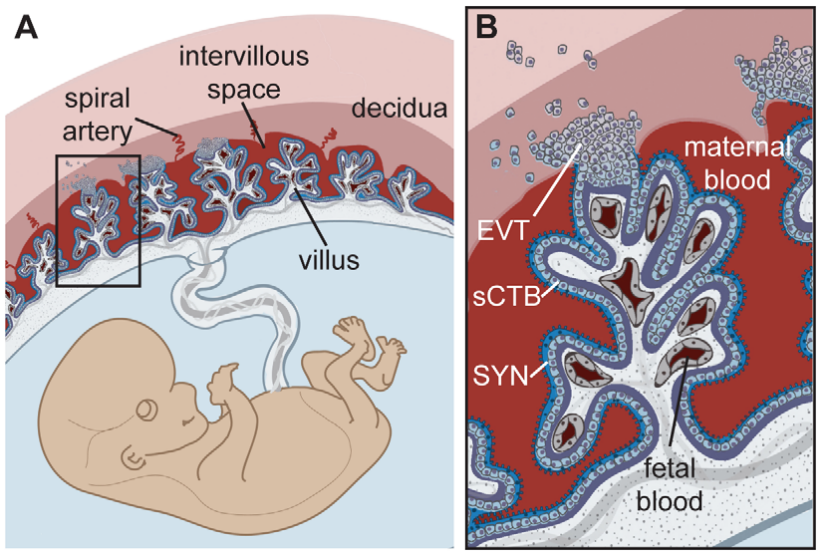
Human placental structure. (**A**) Structure and orientation of fetus and placenta in uterus. The uterine lining transforms into decidua. Maternal blood circulates freely in the intervillous space that contains numerous villous trees that are fetal in origin. (**B**) Enlargement of box in panel A. Maternal blood bathes the villous trees, which are covered by syncytium (SYN) that is underlaid by a population of progenitor cells called subsyncytial cytotrophoblasts (sCTB) and a basement membrane (purple). Fetal capillaries are located in the villous stroma. Exchange of gas and nutrients between maternal and fetal blood occurs across the syncytium. Cytotrophoblasts that come in contact with the decidua differentiate along the invasive pathway into extravillous trophoblasts (EVT). EVT anchor the placenta in the uterus and invade uterine vessels (not shown) to allow maternal blood to flow into the intervillous space.

What are the barriers of the placenta to infection and how is *L. monocytogenes* able to breach them? Understanding the structure of the human placenta is critical for addressing these questions. The placenta is comprised of maternal and fetal cells (Fig. 1) [17]. Prior to implantation, the maternal uterine lining transforms into the receptive decidua. Shortly after, specialized fetally derived cells called trophoblasts differentiate into several subpopulations that perform critical placental functions. Specifically, invasive extravillous trophoblasts (EVT) anchor the placenta in the uterus and invade the decidua and maternal spiral arteries. Consequently, maternal blood flows into the intervillous space, bathing the fetally derived villous trees. These villi are covered by a continuous layer of multinucleate syncytium (SYN), a specialized trophoblast layer that mediates gas, nutrient and waste exchange between mother and fetus. The syncytium is underlaid by progenitor cells called subsyncytial cytotrophoblasts (sCTB), which are separated by a basement membrane from the stroma of the villi where fetal capillaries are found.

We and others have previously shown that primary human placental organ cultures are relatively resistant to infection with *L. monocytogenes* [18], [19]. We utilized organ cultures from first trimester placentas to define where and how *L. monocytogenes* breaches the maternal-fetal barrier [19]. The syncytium that is in direct contact with maternal blood is highly resistant to infection. The other maternal-fetal interface is in the decidua where invasive EVT are in direct contact with maternal cells and tissues. Even though this interface has a much smaller surface area, it is the preferred site of initial placental infection. Indirect evidence suggested that EVT are capable of restricting intracellular growth and cell-to-cell spread of *L. monocytogenes* [19]. In this study, we further characterize the intracellular fate of *L. monocytogenes* in EVT.

We used a cell culture model system of primary human EVT [20] to understand how these specialized cells are able to delay or inhibit the intracellular life cycle of *L. monocytogenes*. We found that isolated EVT were able to restrict intracellular bacterial growth and spread. Furthermore, EVT prevented vacuolar escape and steered vacuolated bacteria towards degradation in lysosomes. This phenotype was strongest in invasive EVT; cells that *in vivo* are in contact with maternal tissues. Our results suggest that EVT have effective defense mechanisms against intracellular pathogens and form a significant bottleneck in the transplacental transmission of pathogens.

## Results

### Culture of primary EVT

In order to examine the role of EVT in placental infection, we characterized the intracellular fate of *L. monocytogenes* in isolated primary human EVT. We used a well-established model system that has been previously used to study differentiation of progenitor cytotrophoblasts along the invasive pathway [21]. In this cell-culture system, cytotrophoblasts are isolated from second trimester placentas and induced towards differentiation along the invasive phenotype by culture on extracellular matrix such as Matrigel [22]. Tissue from the second trimester is used because the yield of cytotrophoblasts is higher than from first and term placentas [20]. Cytotrophoblasts are isolated by a series of enzymatic digestions and ficoll gradients. In order to assure a pure population of cytotrophoblasts, we performed a CD45-based depletion to remove any remaining immune cells prior to culture and differentiation. Cytokeratin 7, a cytotrophoblast marker [23], was used to determine the purity of the cell population. Generally our cell preparations contained over 95% cytotrophoblasts, with the remaining <5% being predominantly placental fibroblasts from the villous stroma (data not shown).

### Intracellular fate of *L. monocytogenes* in EVT

**Figure 2:**
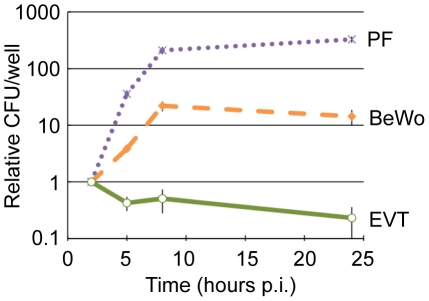
EVT restrict intracellular growth of *L. monocytogenes*. Intracellular growth curves of wild type *L. monocytogenes* in 3 cell types: primary placental fibroblasts (PF), choriocarcinoma cell line (BeWo), and primary extravillous trophoblasts (EVT). CFU/well were normalized to the 2-hour time point within each experiment. Each data point is an average of 3 independent experiments for PF and BeWo, and 10 independent experiments for EVT. Bars represent SEM.

EVT were infected with wild type *L. monocytogenes* at a multiplicity of infection (MOI) of 5. Gentamicin was added to the culture medium at 1 hour post-inoculation (p.i.) to eliminate extracellular bacteria. We observed a ∼100-fold variation in susceptibility to infection across EVT from different placentas: 0.1–12% of EVT were infected, on average with one bacterium, at 2 hours p.i. This may be due to genetic differences between donors or heterogeneity in the condition of the placentas. We therefore normalized the growth of bacteria over time to the number of bacteria at the 2-hour time point for each placenta. Normalized data from 10 individual donor placentas was averaged to minimize the effects of individual differences. In contrast to almost all other previously studied cell types—which generally support *L. monocytogenes* growth [9]—intracellular bacteria in EVT decreased 2-fold between 2 and 5 hours p.i. and continued to decrease by 5-fold over the next 19 hours (Fig. 2).

For comparison, the three commercially available human trophoblast-derived choriocarcinoma cell lines (BeWo, Jeg3, and Jar) were also infected at an MOI of 5, which resulted in significantly greater infection: ∼14% of cells at 2 hours p.i. in BeWo (p = 0.004 by Student's T-test). Invasion of EVT and BeWo cells is InlA-dependent [18], [19], [24]. We observed lower levels of E-cadherin expression by immunofluorescence microscopy, the host cell receptor for InlA, in isolated EVT in comparison to BeWo cells (data not shown), which most likely accounts for the difference in invasion between these two cell types.

In all of the choriocarcinoma cell lines *L. monocytogenes* grew with a similar doubling time of about 77 minutes between 2 and 5 hours p.i. (Fig. 2 and data not shown). This is 2-fold slower than intracellular growth rates in murine macrophage cell lines [25], and in sharp contrast to the “halving time” of *L. monocytogenes* in EVT of about 310 min (p<10^−4^ by Student's T-test). In addition, we tested the fate of *L. monocytogenes* in primary human placental fibroblasts. These fibroblasts were isolated from a first trimester placenta [26] and propagated in culture for at least 10 generations before infection to ensure purity. To compare with EVT, placental fibroblasts were infected at an MOI of 60, which resulted in infection of ∼1% of cells at 2 hours p.i. Subsequently, *L. monocytogenes* grew with a doubling time of 40 minutes between 2 and 5 hours p.i. (Fig. 2).

### Escape of *L. monocytogenes* into the cytosol

*L. monocytogenes* grows rapidly in the host cell cytosol [9], while mutants that are unable to access the cytosol generally do not replicate [25]. Therefore, the lack of intracellular growth of *L. monocytogenes* in EVT could be due to an inability to escape from the primary vacuole. We thus determined whether *L. monocytogenes* can escape from the primary vacuole in EVT and whether bacteria can be found in the host cell cytosol.

To determine vacuolar escape, we utilized a *L. monocytogenes* strain that expresses red fluorescent protein (RFP) under the *actA* promoter (p*actA*-RFP). ActA nucleates actin, and its transcription is up regulated 200-fold in the host cell cytosol [27], [28]. Therefore, the expression of RFP in this strain correlates with entry of bacteria into the cytoplasm [29]. In addition, polymerization of host actin filaments around bacteria indicates cytosolic localization of *L. monocytogenes* and can be visualized by staining fixed cells with fluorescently labeled phalloidin, a compound that binds F-actin [30].

**Figure 3:**
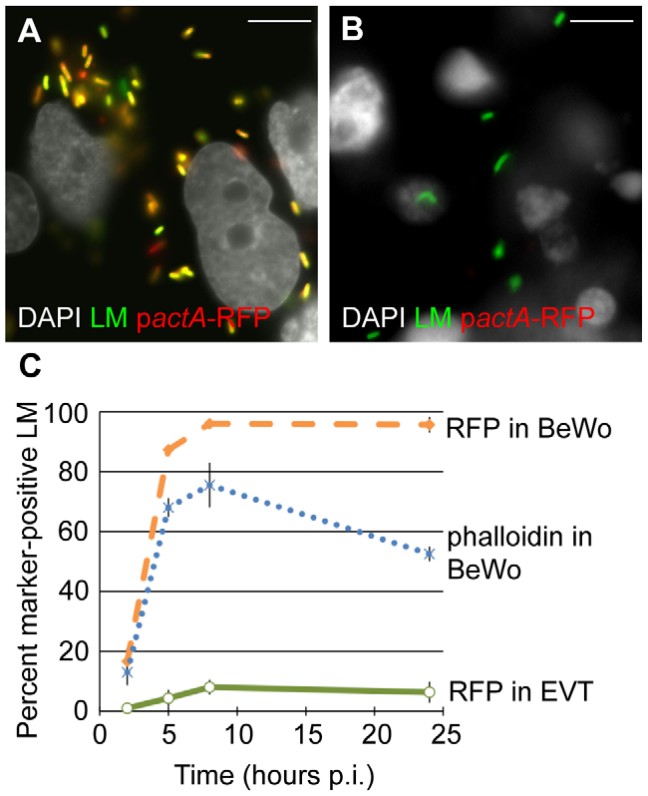
Vacuolar escape of *L. monocytogenes* (LM) is strongly impaired in EVT. BeWo cells and EVT were infected with p*actA*-RFP LM and counterstained with anti-LM antibody (green). (**A,B**) Representative images of BeWo (**A**) and EVT (**B**) 8 hours post-inoculation (p.i.). DAPI counterstain (white). Scale bar is 10 µm. (**C**) Percent RFP-expressing bacteria were enumerated and represent bacteria that escaped from the primary vacuole. Actin nucleation by wild type LM in BeWo cells was measured by co-localization with phalloidin. Bacterial RFP expression and phalloidin-co-localization are expressed as a percentage of the total number of intracellular bacteria. Each data point is an average of 3 independent experiments. Bars represent SEM.

First we analyzed vacuolar escape rates of *L. monocytogenes* in BeWo cells. We infected BeWo cells with p*actA*-RFP *L. monocytogenes* and fixed the cells for immunofluorescence microscopy at 2, 5, 8 and 24 hours. The preparation was counterstained with polyclonal anti-*Listeria* antibody to visualize the total number of bacteria per cell. Microscopic inspection of BeWo cells at 8 hours p.i. showed that the vast majority of bacteria expressed RFP (Fig. 3A). The percentage of RFP-expressing bacteria increased from 17% to 95% between 2 and 8 hours p.i (Fig. 3C). Consistent with high vacuolar escape rates in this cell line, the number of bacteria that co-localized with phalloidin steadily increased from 13% to 76% over the same time period (Fig. 3C). Between 8 and 24 hours p.i. RFP expression remained at 95%, consistent with the long half-live of RFP [31], which led to RFP persistence in all bacteria that had escaped the primary vacuole. In contrast, phalloidin co-localization decreased to 53% at 24 hours p.i. The significant difference between RFP expression and phalloidin co-localization at 8 hours p.i (p = 0.04 by Student's T-test) and the observed decrease in phalloidin co-localization at 24 hours p.i. is most likely due to the fact that phalloidin staining provides a snapshot of intracellular bacteria that are in the actin-nucleating stage of their life cycle. In contrast to RFP expression, phalloidin does not co-localize with bacteria that have spread to neighboring cells and are still in the secondary vacuole. It is unlikely that host cell death at 24 hours p.i. contributes substantially to the decrease in phalloidin co-localization, because host cell death would lead to a significant decrease in intracellular bacteria as well [32], which we do not observe (Fig. 2).

In contrast, infection of EVT with p*actA*-RFP *L. monocytogenes* counterstained with polyclonal anti-*Listeria* antibody revealed that the vast majority of bacteria did not express RFP at 8 hours p.i. (Fig. 3B). Quantitation showed that less than 10% of *L. monocytogenes* expressed RFP over the 24-hour course of infection (Fig. 3C), compared to nearly 100% in BeWo cells (p<10^−5^ by Student's T-test). In addition, no co-localization of *L. monocytogenes* with phalloidin was observed in EVT (data not shown). Our results suggest that bacteria are unable to grow in EVT because they are trapped in the primary vacuole.

In the murine model of infection the virulence factor LLO, a cholesterol-dependent pore-forming cytolysin, is essential for vacuolar escape [33]. We evaluated the role of LLO in the intracellular fate of *L. monocytogenes* in EVT. We tested two bacterial strains: DP-L2161 which is deficient in LLO [34] and unable to grow in BeWo cells (data not shown) and DP-L4057 which has a mutation in LLO (S44A) that increases phagosomal escape in murine bone marrow derived macrophages [35]. The outcome of infection did not differ from wild type infection - both strains were eliminated in EVT over 24 hours - although with slightly different kinetics (see Supplementary Fig. S1). These results suggest the possibility that LLO function is impaired in EVT.

### The *L. monocytogenes*-containing vacuole in EVT

The maturation of *L. monocytogenes*-containing vacuoles has been studied in detail in murine macrophage cell lines (RAW 264.7 and J774A.1) [12]. Wild type *L. monocytogenes* escapes from a vacuolar compartment that includes the late endosomal marker Rab7. The early endosomal marker Rab5 does not associate with *L. monocytogenes* even at very early time points after phagocytosis. If the vacuole matures further and acquires the lysosomal marker Lamp1, the rate of vacuolar escape is minimal.

**Figure 4:**
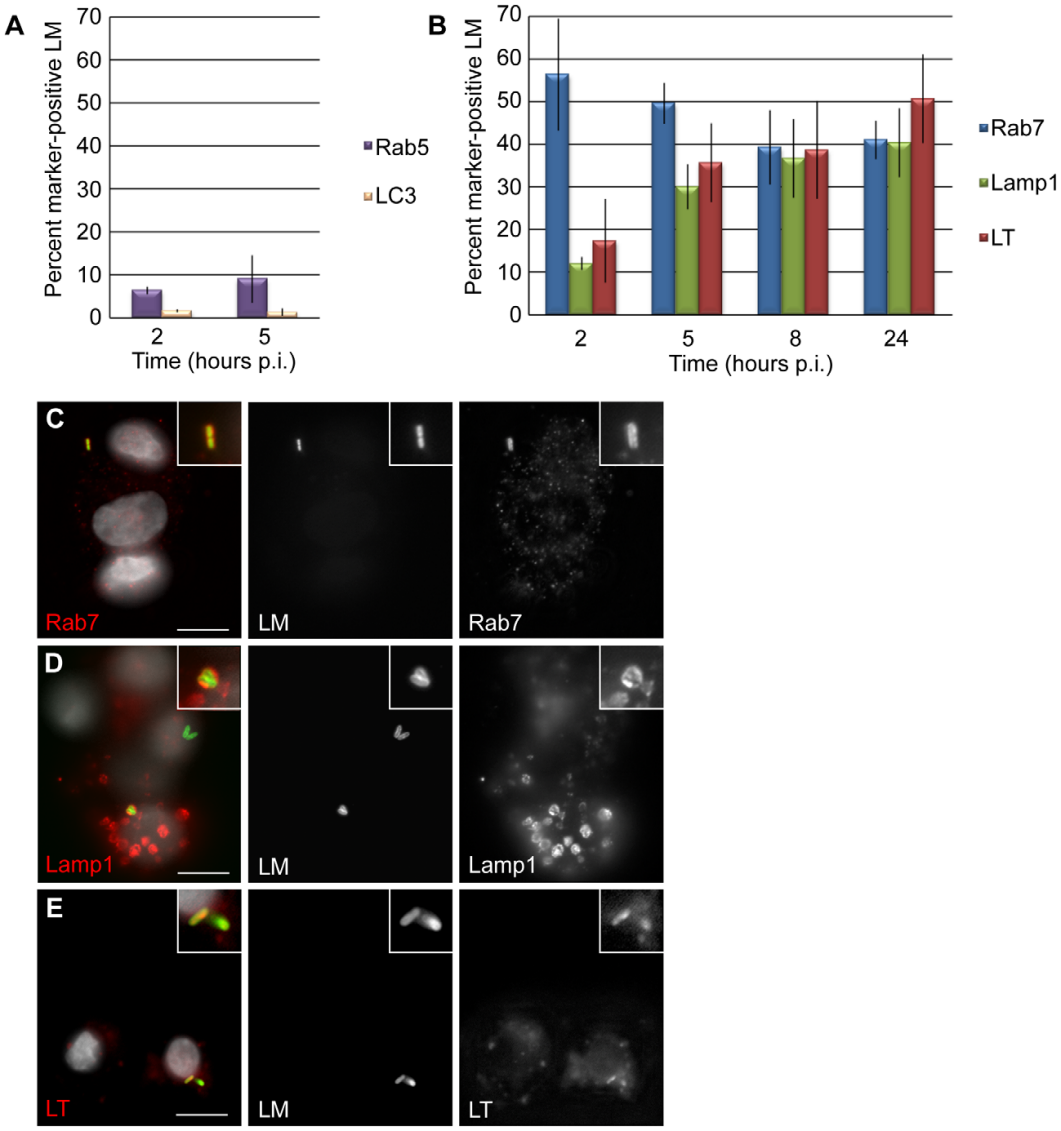
*L. monocytogenes* (LM) is trapped in late endosomes and acidified lysosomes in EVT. (**A**) Percent co-localization of early endosomal marker Rab5 and autophagosomal marker LC3 with LM. (**B**) Percent co-localization of late endosomal marker Rab7, lysosomal marker Lamp1 and acidotropic dye Lysotracker (LT) with LM. p.i.  =  post-inoculation. Each data point is an average of 3 independent experiments. Bars represent SEM. (**C–E**) Representative images of GFP-expressing LM (green) co-localizing with markers Rab7, Lamp1, and LT (red). The color-merged images also show DAPI counterstain (white). Scale bars are 10 µm.

To characterize the vacuolar compartment that *L. monocytogenes* occupies in EVT, we examined these same markers. The early endosomal marker Rab5 was associated with less than 10% of bacteria at 2 and 5 hours p.i. (Fig. 4A). The late endosomal marker Rab7 was found to co-localize with 55% of bacteria at 2 hours p.i. and with over 40% of bacteria at all other time points through 24 hours of infection (Fig. 4B,C). Lamp1 co-localized with only 12% of bacteria at 2 hours p.i. and increased steadily to 40% at 24 hours p.i. (Fig. 4B,D).

To test whether the *L. monocytogenes*-containing vacuole in EVT becomes acidified, we used the acidotropic dye Lysotracker. Lysotracker staining followed a similar trend to Lamp1 staining: 17% of bacteria were found in an acidified compartment at 2 hours p.i., increasing to 51% at 24 hours p.i. (Fig. 4B,E).

While it is generally believed that *L. monocytogenes* replicates in the cytoplasm and not in vacuoles, there have been a few reports suggesting the possibility of slow replication in vacuolar compartments. Bhardwaj et al. described the presence of multiple bacteria in membrane-bound vacuoles in mononuclear cells in the liver of SCID mice with chronic listeriosis [36]. Furthermore, Birmingham et al. found that 13% of bacteria in a murine macrophage cell line were replicating slowly in autophagosome-like vacuolar compartments (LC3-positive, LAMP1-positive, non-acidified) and named these structures SLAPS (spacious *Listeria*-containing autophagosomes) [37]. We therefore evaluated whether *L. monocytogenes* co-localizes with the autophagy marker LC3, but found little to no co-localization in our system (Fig. 4A). We concluded that bacteria in EVT are trapped in vacuoles that mature into acidified lysosomes, suggesting that *L. monocytogenes* is degraded in this compartment.

### Vacuolar localization of *L. monocytogenes*

**Figure 5:**
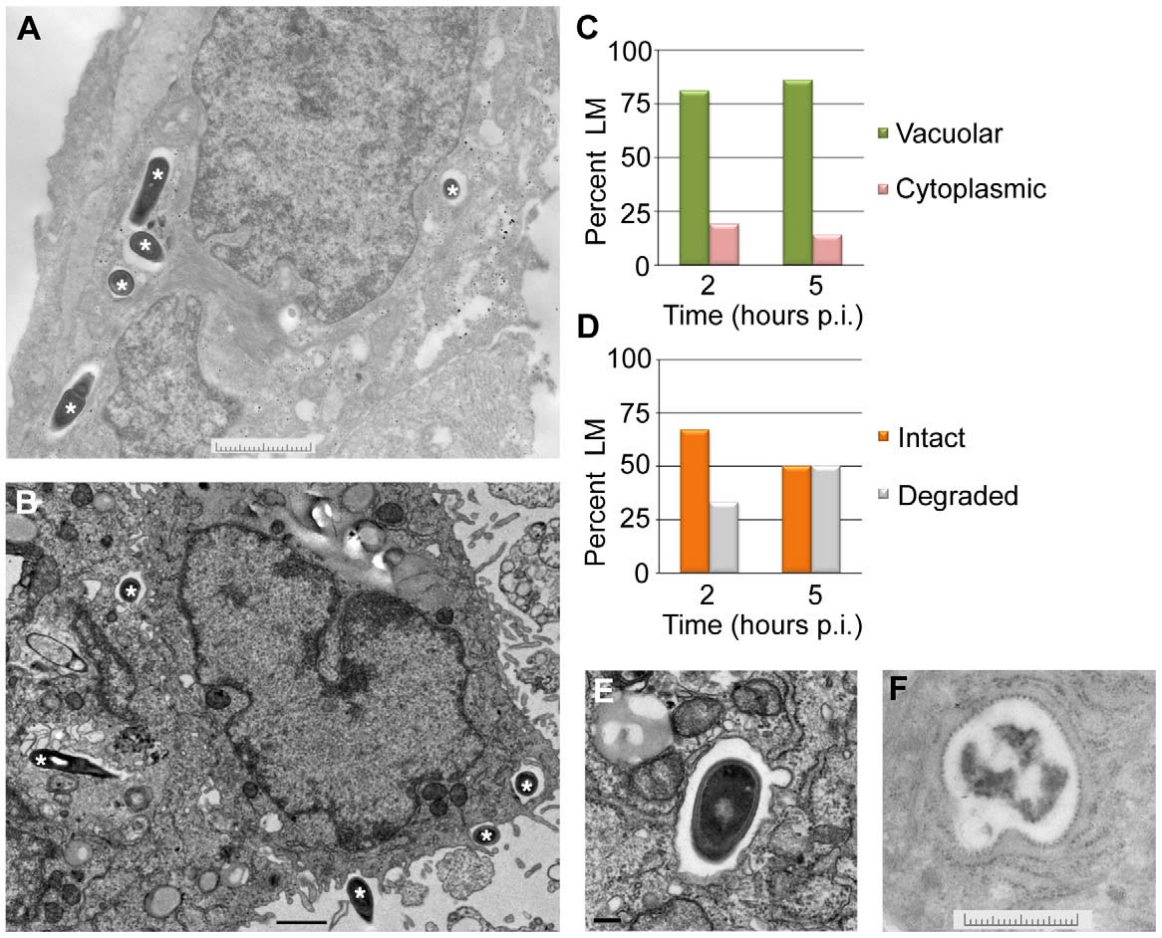
*L. monocytogenes* (LM) in EVT is largely confined to vacuoles. (**A,B**) Representative transmission electron micrographs from 2 (**A**) and 5 (**B**) hours post-inoculation (p.i.). Bacteria are marked with asterisks. Scale bars are 2 and 1 µm respectively. (**C**) Quantification of subcellular localization of bacteria in EVT at 2 and 5 hours p.i. (**D**) Quantification of intact versus degraded bacteria in vacuoles in EVT at 2 and 5 hours p.i. (**E**) Close-up image at 5 hours p.i. shows a single membrane vacuole surrounding intact bacterium. Scale bar is 100 nm. (**F**) Close-up image of degraded bacterium in a vacuole in EVT at 5 hours p.i. Scale bar is 2 µm.

To look more closely at the subcellular localization of *L. monocytogenes* in EVT, transmission electron microscopy was performed. EVT were infected with wild type *L. monocytogenes* at an MOI of 60. This high inoculum was used to increase the number of infected cells and the number of bacteria/cell for better visualization. Because the most significant decrease in intracellular bacterial numbers occurred between 2 and 5 hours p.i. (Fig. 2), infected EVT at those time points were examined (Fig. 5A,B). The number of vacuolar *L. monocytogenes* was enumerated: at both time points, 81–86% of bacteria were confined to vacuoles (Fig. 5C). These escape rates (14–19%) are slightly higher than those measured using the p*actA*-RFP strain above. This difference is significant (p = 0.004 by Student's T-test) and is likely due to differences in the infection (MOI of 5 versus 60) and/or due to a more limited detection threshold of RFP fluorescence as compared to electron microscopy. Furthermore, we enumerated the number of bacteria that appeared intact versus degraded. Intact appearing bacteria decreased from 67% to 50% between 2 and 5 hours p.i., and degraded bacteria increased from 33% to 50% during the same time interval (Fig. 5D,E,F).

A vacuolar compartment derived from the primary vacuole consists of a single lipid bilayer, whereas secondary vacuoles (a result of infection via cell-to-cell spread) and autophagosomes typically consist of two lipid bilayers [15], [38]. With a membrane contrast-enhancing stain and at higher magnification the membranes of the *L. monocytogenes*-containing vacuoles were visualized and appeared to consist of a single lipid bilayer (Fig. 5E). These ultrastructural results are consistent with bacterial entrapment in the primary vacuole and degradation in lysosomes.

### Vacuolar escape of *L. monocytogenes* in placental explants

**Figure 6:**
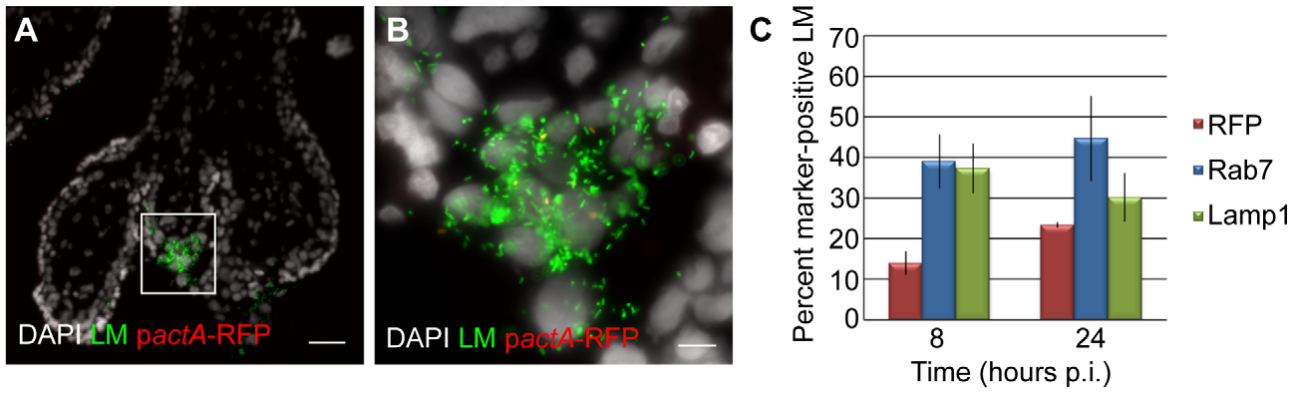
*L. monocytogenes* (LM) is trapped in late endosomes and lysosomes in EVT of placental explants. (**A**) Representative image of placental explant 8 hours post-inoculation (p.i.) with p*actA*-RFP LM counterstained with anti-LM antibody (green). DAPI counterstain (white). Scale bar is 50 µm. (**B**) Close-up of boxed area in panel A. Scale bar is 10 µm. (**C**) Vacuolar escape measured by percent RFP expression of p*actA*-RFP strain counterstained with anti-LM antibody (green). Percent co-localization of LM with late endosomal marker Rab7 and lysosomal marker Lamp1. Each data point is an average of 3 independent experiments. Bars represent SEM.

We previously found that EVT are the preferred initial site of infection for *L. monocytogenes* in first trimester placental organ cultures [19]. Furthermore, we observed that *L. monocytogenes* is able to spread beyond the EVT along sCTB in some placentas. Under the conditions Robbins et al. used, such spread occurs in 50% of placentas over a time period of 72 hours. The inability of *L. monocytogenes* to escape from the primary vacuole in EVT could explain the delay or lack of listerial dissemination in placental organ cultures. Thus, we analyzed the rates of vacuolar escape in first trimester placental organ cultures infected with p*actA*-RFP *L. monocytogenes* and counterstained with polyclonal anti-*Listeria* antibody as described above (Fig. 6A,B). At 8 hours p.i., only 14% of bacteria had escaped the vacuole, while 39% and 37% were in late endosomes and lysosomes respectively (Fig. 6C). By 24 hours p.i. the percentage of RFP-expressing bacteria increased to 23% and the proportion of *L. monocytogenes* co-localizing with Rab7 and Lamp1 remained around 40% (Fig. 6C).

Vacuolar escape rates in placental organ cultures were somewhat higher than those observed in isolated second trimester EVT (Fig. 3C; p = 0.19 by Student's T-test). We therefore decided to test whether vacuolar escape rates differ between distinct trophoblast subpopulations. When isolated cytotrophoblasts are grown on Matrigel they differentiate along the invasive pathway and therefore consist of a more homogeneous EVT population [20], [21]. In contrast, there are several distinct subpopulations of trophoblasts *in vivo* and *ex vivo* that are in different stages of differentiation ranging from progenitor cytotrophoblasts near the stroma to invasive EVT at the outer villus margin. Therefore, infection of placental organ cultures leads to infection of a mixed population of trophoblasts.

**Figure 7:**
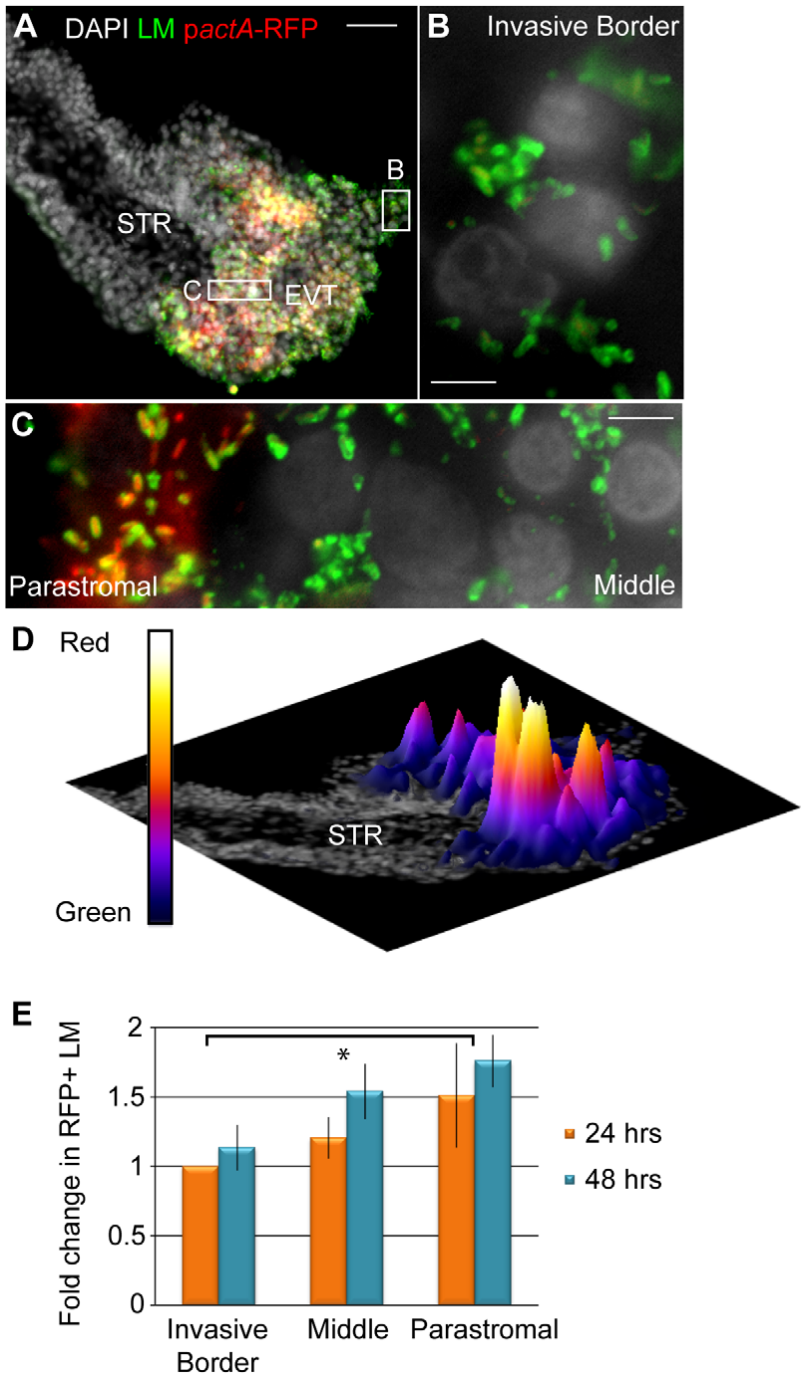
*L. monocytogenes* (LM) vacuolar escape rates vary across subpopulations of trophoblasts. (**A**) Representative image of placental explant 24 hours post-inoculation (p.i.) with p*actA*-RFP LM counterstained with anti-LM (green). DAPI counterstain (white). STR  =  stroma. (**B,C**) Close-up examples of Invasive Border EVT (**B**), Parastromal trophoblasts and middle EVT (**C**). (**D**) Rotated image of placental explant from panel A with superimposed surface plot representing areas of highest RFP expression in relation to total bacterial green signal. Highest peaks are in the parastromal trophoblast region, where vacuolar escape rates are highest. (**E**) Percent RFP-expressing bacteria in each trophoblast subpopulation were enumerated at 24 and 48 hours p.i. in villi where all 3 subpopulations were infected. RFP expression was normalized to the 24-hour time point in invasive border EVT from the same placenta. Results from 3 independent experiments were expressed as average fold-change in comparison to the escape rate in invasive border EVT at 24 hours. Bars are SEM. Asterisk represents significant difference (p = 0.02).

To test whether listerial escape rates differ in different trophoblast subpopulations, we compared escape rates in three distinct populations of trophoblasts: (1) trophoblasts that were in contact with Matrigel (invasive border EVT), (2) trophoblasts that were surrounded by other trophoblasts on all sides (middle EVT), and (3) those that were in contact with the basement membrane and its underlying stroma (parastromal trophoblasts) (Fig. 7A–C). We increased the dose of *L. monocytogenes* to 2x10^7^ bacteria/ml for 5 hrs before addition of gentamicin in order to achieve infection of all three subpopulations within one placenta at 24 hours p.i., and compared escape rates between invasive border EVT, middle EVT and parastromal trophoblasts at 24 and 48 hours p.i. (Fig. 7D,E). The average escape rate in invasive border EVT at 24 hours p.i. was 40% (range 11% to 55%). Because of this large variability between placentas from different donors, we normalized the escape rates in middle EVT and parastromal trophoblasts to the escape rate in invasive border EVT from the same placenta. At 24 hours p.i. we determined the fold-difference in escape rates in middle EVT and parastromal trophoblasts in comparison to the escape rate in invasive border EVT from the same placenta. Vacuolar escape rates increased the closer the trophoblasts were to the core of the placental villus. The average increase in escape rates compared to invasive border EVT was 1.21-fold in middle EVT and 1.51-fold in parastromal trophoblasts (Fig. 7E). At 48 hours p.i. we determined the fold difference in escape rates in all three subpopulations in comparison to the escape rate in invasive border EVT at 24 hours p.i., and found similar results. The average increase in escape rates was 1.14-fold (invasive EVT), 1.54-fold (middle EVT), and 1.76-fold (parastromal trophoblasts) (p = 0.02 by Student's T-test for combined 24- and 48-hour time points). We concluded that EVT at the invasive border—a cell type that is in direct contact with maternal cells *in vivo*—are especially prohibitive for listerial vacuolar escape. However, if *L. monocytogenes* is able to spread beyond the invasive EVT it can find a more hospitable environment.

## Discussion

Much of the pioneering work on the *L. monocytogenes* life cycle and intracellular growth kinetics has been performed in murine bone marrow derived macrophages as well as various murine and human cell lines [9], [15], [39]. In these cells, *L. monocytogenes* vacuolar escape rates are 80% or higher [29], [40], and bacteria grow rapidly (generation time of ∼40 min) in the nutrient-rich cytosol [25]. In contrast, the vacuolar escape rates in isolated primary EVT were less than 10%.

It is possible that vacuolar escape and growth rates vary depending on the specific cell type, especially in cells that play a role in host defense against infection. For example, primary murine dendritic cells are less hospitable to *L. monocytogenes* than primary bone marrow-derived mouse macrophages [40], [41]. Westcott et al. showed that bacterial doubling time is about 2-fold slower in primary murine dendritic cells (∼70 min), and only ∼40% of the bacteria escape into the cytosol. Specific endosomal maturation features in dendritic cells that are important for efficient processing and presentation of bacterial antigens to T cells are thought to be the underlying reason for these decreased vacuolar escape rates. Primary murine peritoneal macrophages are even more hostile to *L. monocytogenes*: Portnoy et al. has demonstrated that these cells kill ∼80% of *L. monocytogenes* during the first 2 hours p.i. and that surviving bacteria grow at a generation time of ∼120 min or longer [42]. Furthermore, if resident peritoneal macrophages are stimulated with IFNγ, bacterial growth is eliminated, and 95% of bacteria are found in vacuolar compartments [42]. While these activated professional immune cells are known to be critical in scavenging and containing infectious particles, it is more surprising that epithelial cells in the placenta, the EVT, would possess a similar bacteriocidal phenotype. In this context, it is interesting that IFNγ is crucial for a successful pregnancy and present at high levels at the maternal-fetal interface [43]. IFNγ is produced by uterine natural killer cells, which comprise approximately 20–40% of the leukocytes in the decidua [44], [45], and IFNγ receptors are expressed on human trophoblast cells throughout pregnancy [46]. It is possible that residual effects of *in utero* IFNγ exposure contribute to decreased vacuolar escape and increased bacterial degradation in EVT.

Why are bacteria not able to escape the vacuole in EVT? In the murine model of infection the virulence factor LLO is essential for vacuolar escape [33]. We found that lack of LLO or increased hemolytic activity of LLO did not alter the outcome of infection in EVT, suggesting that LLO function is impaired in this cell type. LLO-mediated pore formation is a pH dependent process, with a pH optimum of 5.5 [47], [48]. Although the *Listeria*-containing vacuole in EVT acidifies, the kinetics or extent of acidification could present unfavorable conditions for LLO function. For example LLO loses its hemolytic activity at neutral pH in less than 10 min [49]. Another possibility is that the *Listeria*-containing vacuole has a different lipid composition that renders LLO non-functional. LLO is dependent on the presence of cholesterol, which is utilized by EVT for the synthesis of progesterone [50]. Specialized hormone synthesis in EVT could lead to differences in cholesterol metabolism and/or distribution in these cells, rendering it inaccessible to vacuolar LLO. Moreover, the active form of a host-derived thiol reductase (GILT) involved in antigen processing has been shown to be required for the activation of LLO [51], and may not be present or accessible in the *Listeria*-containing vacuole in EVT. However, all of the above mentioned studies have been performed in the murine model of infection. In contrast, in many human cell types *L. monocytogenes* deficient in LLO is capable of vacuolar escape [25], [52], [53]. The mechanisms of LLO-independent vacuolar escape are poorly understood [54], but the existence of these examples opens up a myriad of other pathways that may be different in EVT, that ultimately could lead to vacuolar entrapment of *L. monocytogenes*. Further studies will be needed to assess these possibilities.

Work in several pregnant animal models of listeriosis supports our findings that *L. monocytogenes* has to pass several bottlenecks to infect the placenta and spread to the fetus. We have shown previously that the placenta in the pregnant guinea pig model is relatively protected from colonization, characterizing the kinetics of bacterial spread from maternal organs to the placenta and to the fetus [55]. The guinea pig placenta is colonized with 10^4^-fold fewer bacteria than maternal liver and spleen after intravenous inoculation, and the bottleneck between placenta and fetus is again 1∶10^4^ bacteria. Studies in the pregnant mouse and gerbil models also require high intravenous inoculums, >10^6^ bacteria, to induce placental infection [56], [57].

Interestingly, there are several lines of evidence that suggest EVT are a suboptimal niche for the growth of intracellular pathogens in general. Human CMV infection, for example, is inefficient in trophoblasts, progresses slowly, and releases only small amounts of progeny virus [58]. Likewise, placentas infected with CMV *in utero* show rare viral replication in EVT, with membrane-clustered virions [59]. Recent studies with HIV-1 indicate that EVT are also non-permissive to HIV-1 replication due to active degradation and/or passive inactivation of critical viral replication mechanisms [60]. Others have observed that the majority of HIV-1 virions are trapped within endosomal compartments [61]. The common thread in these studies is that vacuolar or endosomal trafficking is hindering the normal life cycles of pathogens and preventing growth and spread of the virus or bacterium. While little is known about EVT in general, ultrastructural studies of uninfected human placentas report many unidentified vesicles and vacuoles in EVT [62], [63]. It is possible that the invasive role of EVT and their active degradation of extracellular matrix may require unique degradative and/or endosomal pathways that interfere with the life cycle of intracellular pathogens. As a result, EVT create a significant barrier to infection, and pathogens must get past the bacteriocidal EVT into more permissive cells in the placenta for the infection to progress.

If the primary site of infection is an inhospitable cell-type, then how does placental infection progress to cause pregnancy complications and fetal infection? One possibility is that even though EVT are the preferred site of initial infection with *L. monocytogenes* [19] and *Toxoplasma gondii* (our unpublished observations), and can harbor CMV *in utero* [64], they are a dead end for pathogens. This seems unlikely because we have not observed placental infection without infection of EVT, and *L. monocytogenes* can spread beyond EVT in some placentas [19]. In addition, other routes of crossing the trophoblast barrier appear even more difficult, since the syncytium is highly resistant to infection with *L. monocytogenes* [19] and *T. gondii* (our unpublished observations). It is possible that EVT could differ in their resistance to infection due to host genotypic differences. This would mean that some people are simply more predisposed to placental infection and pregnancy complications than others. However, to our knowledge no genetic basis for differences in susceptibility to vertical transmission has ever been identified.

We hypothesize that EVT can either contain or eliminate an infection until a certain threshold of cellular damage or placental inflammation is surpassed. For instance, non-infectious pregnancy complications that influence oxygen tension or pH in the placenta could alter the biochemical and/or physiological condition of EVT and decrease their resistance to infection. Co-infection with other pathogens could similarly escalate an immune imbalance at the maternal-fetal interface. If these imbalances threaten the healthy progression of pregnancy, spontaneous abortion or preterm labor are initiated to avoid continuation of pregnancy with a compromised placenta.

The placenta has developed a marvelous defense against infection, most likely consisting of multiple layers of physical and biochemical barriers. Both subpopulations of trophoblasts—syncytium and EVT—that are in direct contact with maternal cells and tissues are effective barriers against infection. The syncytium is in direct contact with maternal blood and is highly resistant to infection. The EVT are in close contact to maternal cells and tissues in the implantation site, and are the preferred initial sites for infection, but are inhospitable to a variety of intracellular pathogens. Both barriers can probably be breached by additional damage, resulting in infection of subsyncytial cytotrophoblasts, which appear to be more hospitable to intracellular replication of pathogens. Nevertheless, *L. monocytogenes* still has to pass another physical barrier: the basement membrane [19], to reach the villous stroma where the fetal capillaries are. *L. monocytogenes* will serve as an excellent model to characterize the precise molecular basis of the maternal-fetal barrier.

## Methods

### Ethics statement

This study was conducted according to the principles expressed in the Declaration of Helsinki. The study was approved by the Institutional Review Board at the University of California, San Francisco, where all experiments were performed (H497-00836-29). All patients provided written informed consent for the collection of samples and subsequent analysis.

### Human tissue collection, cell isolation and culture

All chemicals were purchased from Sigma-Aldrich unless otherwise stated. For human placental organ cultures, placentas from elective terminations of pregnancy (gestational age 4 to 8 weeks) were collected and prepared as previously described [65]. Briefly, fragments from the surface of the placenta were dissected into 1–3 mm tree-like villi, placed on Matrigel (BD Biosciences, San Jose, CA)-coated Transwell filters (Millipore, Bedirica, MA, 30-mm diameter, 0.4 um pore size) and cultured in Dulbecco's modified Eagle-F12 medium (DMEM-F12; 1∶1, vol/vol) supplemented with 20% fetal bovine serum (FBS, Fisher Scientific), 1% L-glutamine and 1% penicillin/streptomycin (Invitrogen, Carlsbad, CA).

For EVT isolation, placentas from elective terminations of pregnancy (gestational age 14 to 24 weeks) were collected and prepared as previously described [21], [66]. Briefly, placentas from normal uncomplicated pregnancies were obtained immediately after aspiration and subjected to a series of enzymatic digestions followed by purification over a Percoll gradient. Remaining leukocytes were removed using a magnetic-bead-based EasySep CD-45 Depletion Kit with RoboSep device (Stem Cell Technologies, Vancouver, Canada). For growth curves, purified cells were plated on Matrigel-coated Transwell filters (Millipore, Bedirica, MA, 12-mm diameter, 0.4 um pore size) in serum-free DMEM-high glucose, with 2% Nutridoma (Roche Diagnostics, Indianapolis, IN), 1% L-glutamine, 1% sodium pyruvate, 1% 25 mM HEPES, 1% penicillin/streptomycin at a concentration of 1.25×10^5^ cells/transwell. For immunofluorescence microscopy, purified cells were plated on Matrigel-coated 6-well plates at a concentration of 2×10^6^ cells/well.

Placental fibroblasts were isolated as described [26] from a placenta at gestational age 8 weeks, and were cultured in DMEM-high glucose with 10% FBS, 18% M-199, 1% penicillin/streptomycin. For growth curves and immunofluorescence microscopy, cells were plated on glass coverslips in 24-well plates at 2.5×10^5^ cells/well.

### Cell lines

The choriocarcinoma cell line BeWo (ATCC CCL-98) was cultured in Ham's F12 medium with 10% FBS, 1% L-glutamine, 0.15% sodium bicarbonate, 1% penicillin/streptomycin. For growth curves and immunofluorescence microscopy, cells were plated on glass coverslips in 24-well plates at 2.5×10^5^ cells/well.

### Pathogen strains and growth conditions

*L. monocytogenes* 10403S expressing green fluorescent protein (GFP) (strain DH-L1252) was a gift from Darren Higgins [67]. The p*actA*-RFP strain (PL512) was constructed as follows: The ORF encoding TagRFP from *Entacmaea quadricolor* [31] was codon optimized for expression in *L. monocytogenes* using Gene Designer software [68] and the gene was synthesized *de novo* (DNA2.0, Menlo Park, CA). The synthetic gene was cloned downstream of the *actA* promoter in the vector pPL2 and stably integrated at the *tRNA* ^Arg^ locus of the bacterial chromosome in the wild type *L. monocytogenes* strain DP-L4056 as described previously [69]. Molecular constructs were confirmed by DNA sequencing. For infections, bacteria were grown overnight to stationary phase in BHI (Brain Heart Infusion broth) at 30°C and washed once with PBS before dilution and infection.

### *L. monocytogenes* infection

Cells were incubated in antibiotic-free medium for 1 hr before infection. Bacteria were added for 30 minutes, followed by three washes with PBS and addition of antibiotic-free medium. For CFU (colony forming units) determination gentamicin (50 µg/ml) was added at 60 minutes p.i. EVT were inoculated with 3×10^6^ bacteria/ml (MOI 5), and placental fibroblasts with 4×10^7^ bacteria/ml (MOI 60). At indicated times, cells were lysed with distilled water, aliquots were plated on BHI agar plates, and CFU were enumerated. Infection for immunofluorescence microscopy was performed as outlined above with following modification: at 60 minutes p.i. Matrigel was dissolved by incubation with BD Cell Recovery Solution (BD Biosciences, San Jose, CA) for 40 minutes, and cells were re-plated on fresh Matrigel on Transwell filters in media containing gentamicin (50 µg/ml). Therefore, gentamicin was added at 1 hour 45 minutes p.i. to infected cells that were analyzed by immunofluorescence microscopy. CFU after exposure to the enzymatic solution and gentamicin addition at 1 hour 45 min were not significantly different from those under standard CFU (gentamicin at 1 hour p.i.) conditions (data not shown). For electron microscopy, EVT were infected as above with the following alteration: the infectious dose was 4×10^7^ bacteria/ml (MOI 60). Infection of placental explants was performed as previously described [19] with the following alteration: the infectious dose was lowered to 3×10^6^ bacteria/ml for 30 minutes.

### Immunofluorescence

Explants were fixed in 3% paraformaldehyde, passed through a sucrose gradient and snap-frozen in OCT (Ted Pella, Redding, CA). Histological slicing was performed on a Hacker-Slee cryostat. Glass slides with sections were incubated in acetone, soaked in blocking solution (1% bovine serum albumin (BSA) in PBS), then incubated with primary antibodies, rinsed in PBS, incubated with secondary antibodies, and affixed over Vectashield mounting medium with DAPI (Vector Laboratories, Burlingame, CA).

Cultured cell lines and EVT were fixed in 3% paraformaldehyde. For Lysotracker visualization, the dye was added to cells for 30 minutes at 5 µM and washed in PBS before fixation. For Rab7 staining, cells were rinsed in glutamate lysis buffer (25 mM HEPES, 25 mM potassium chloride, 2.5 mM magnesium acetate, 5 mM EGTA, 150 mM K-glutamate), dipped into liquid nitrogen, rinsed in lysis buffer, and fixed in paraformaldehyde. Transwell filters were cut out of wells, blocked and permeabilized in 1% BSA and 0.1% Triton-X100, then stained as described above in BSA/TritonX-100/PBS solution.

Primary antibodies: polyclonal rabbit *Listeria* O antiserum (1∶1000 BD Biosciences, San Jose, CA), mouse polyclonal Lamp1 antiserum (1∶100 DSHB at University of Iowa), mouse monoclonal Rab5 antibody (1∶100, BD Biosciences, San Jose, CA), mouse monoclonal LC3 antibody (1∶100, gift from Dr. Jay Debnath), rabbit monoclonal Rab7 antibody (1∶1000, gift from Dr. Suzanne Pfeffer). Secondary antibodies: Alexa Fluor 594 goat anti-mouse IgG (1∶500, Invitrogen), Alexa Fluor 488 and 594 goat anti-rabbit IgG (1∶1000 & 1∶500, Invitrogen).

Slides were viewed using an inverted TE2000-E microscope (Nikon, Tokyo, Japan) equipped with a 12-bit cooled CCD camera (Q imaging, Surrey, Canada). Images were collected using Simple PCI software (Hamamats, Sewickley, PA).

### Transmission electron microscopy

For the 2-hour time point, cells were fixed overnight at 4°C in 3% glutaraldehyde, 1% paraformaldehyde in 0.1 M cacodylate buffer. Fixed cells were post-fixed with 2% osmium tetroxide, dehydrated in ethanol and embedded in Epon. Thin sections (70 nm) were cut using a Leica Ultracut-UCT Microtome (Leica Microsystems, USA). Observations were made under a Philips Tecnai 10 transmission electron microscope (Department of Pathology, UCSF), and digital acquisition was performed with a CCD camera (Maxim DL Software, Cyanogen, Canada). For the 5-hour time point, cells were fixed as above, and post-fixed with 1% osmium tetroxide and 1.6% potassium ferrocyanide, stained with 5% uranyl acetate solution, dehydrated with ethanol and embedded. Sections were cut using a microtome (RMC MTX, Reichert Ultracut E, RMC MT6000) and observations made under a Philips Tectani 12 transmission electron microscope (EM lab, UC Berkeley). For quantification, 100 bacteria at each time point were counted and categorized by cytoplasmic versus vacuolar localization and intact versus degraded bacteria.

### Image processing

Images were prepared using ImageJ (RSB, Bethesda, MD), Photoshop and Illustrator (Adobe, San Jose, CA). RGB hues were linearly adjusted but no non-linear alterations were performed.

## Supporting Information

Figure S1 **Infection of EVT with** ***L. monocytogenes*** **strains containing mutations in LLO.** Intracellular fate of mutant versus wild type (WT) *L. monocytogenes* strains in EVT. LLO-minus strain is deficient in LLO (LLO-), and strain S44A has increased hemolytic activity in comparison to wild type LLO. CFU/well were normalized to the 2-hour time point within each experiment. Each data point is an average of multiple independent experiments: WT: n = 10, LLO-: n = 5, S44A: n = 2. Bars represent SEM.
(TIF)
