# Supplementary figures and images for: Invasive Extravillous Trophoblasts Restrict Intracellular Growth and Spread of Listeria monocytogenes
Source: PLoS Pathog. 2011 Mar 3;7(3):e1002005. doi: 10.1371/journal.ppat.1002005 (PMC3048367; doi:10.1371/journal.ppat.1002005)

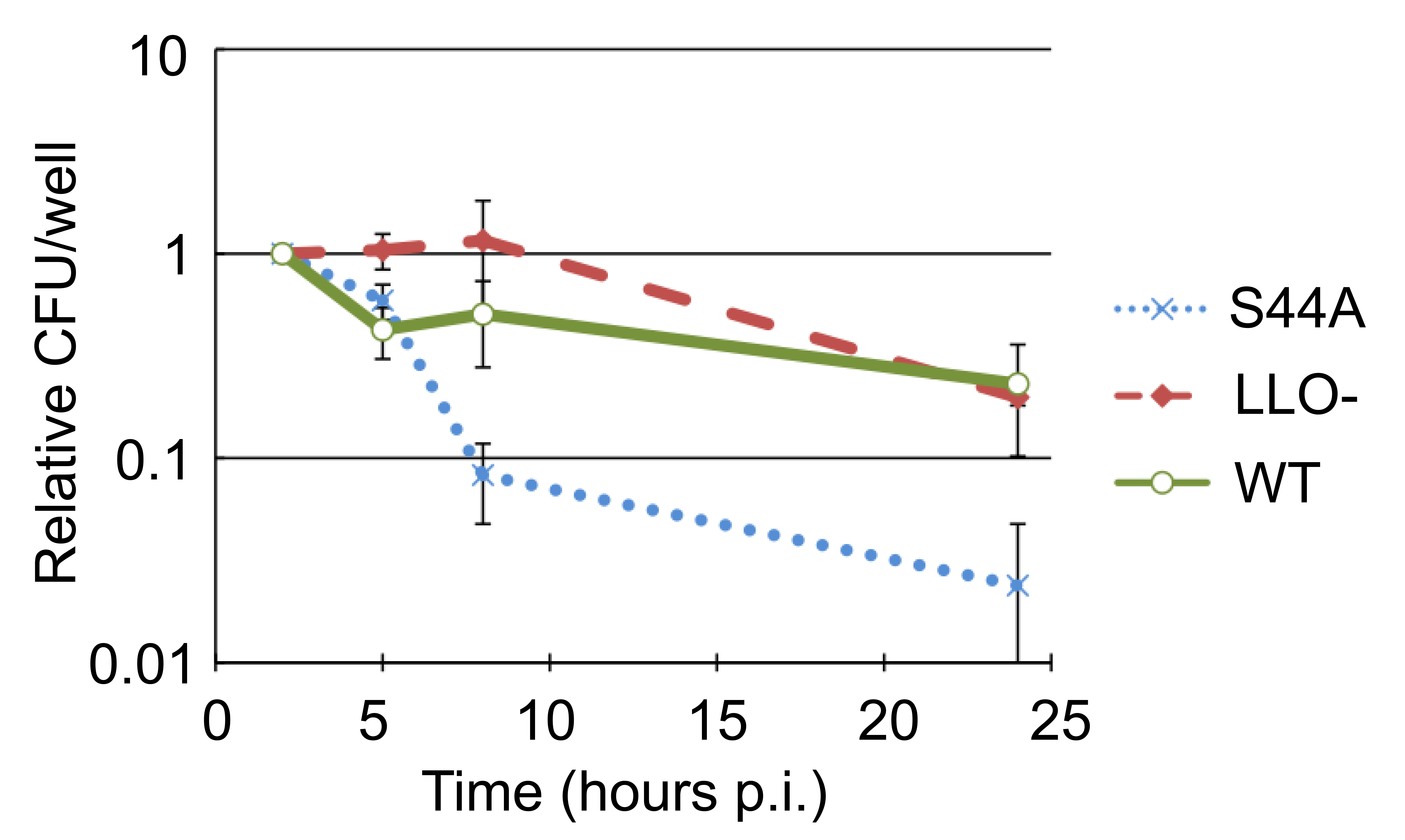

Supplement: Figure S1 — Infection of EVT with L. monocytogenes strains containing mutations in LLO. Intracellular fate of mutant versus wild type (WT) L. monocytogenes strains in EVT. LLO-minus strain is deficient in LLO (LLO-), and strain S44A has increased hemolytic activity in comparison to wild type LLO. CFU/well were normalized to the 2-hour time point within each experiment. Each data point is an average of multiple independent experiments: WT: n = 10, LLO-: n = 5, S44A: n = 2. Bars represent SEM. (TIF) [file ppat.1002005.s001.tif]
